# Employee preferences in health plan design: results from a national survey

**DOI:** 10.1093/haschl/qxag120

**Published:** 2026-06-19

**Authors:** Robert M Kaplan, Margaret C Nikolov, Jeffrey Pfeffer, Arnold Milstein, Sara J Singer

**Affiliations:** Clinical Excellence Research Center, Stanford University School of Medicine, Stanford, CA 94305, United States; Clinical Excellence Research Center, Stanford University School of Medicine, Stanford, CA 94305, United States; Stanford University Graduate School of Business, Stanford, CA 94305, United States; Clinical Excellence Research Center, Stanford University School of Medicine, Stanford, CA 94305, United States; Clinical Excellence Research Center, Stanford University School of Medicine, Stanford, CA 94305, United States; Stanford University Graduate School of Business, Stanford, CA 94305, United States

**Keywords:** employer-Sponsored insurance, consumer preference, health care costs, employee benefits

## Abstract

**Introduction:**

Employer-sponsored health insurance covers over 60% of the U.S. population under age 65 and represents a significant expense for employers, yet employees’ views on benefit design remain understudied.

**Methods:**

We conducted a nationally representative survey of 1200 U.S. adults using employer-sponsored insurance to assess preferences for benefit trade-offs, including narrower provider networks. The survey used stratified sampling and weighting to ensure demographic representativeness.

**Results:**

Nearly half (49.9%) preferred retaining the current balance of salary and benefits. However, 45.9% would accept a narrower network in exchange for higher compensation, and 30.9% supported narrow networks if savings were redirected to other benefits. Trade-off preferences varied by age, sex, race, parental status, and political ideology. Most respondents (74.8%) trusted employers to select plans and believed any cost reductions should be returned to employees (72.0%). Many also supported excluding high-cost providers without demonstrated quality advantages.

**Conclusion:**

The findings suggest that employees are open to cost-efficient plan designs if savings are shared transparently, although other data suggest that few employers are doing this.

Approximately 165 million nonelderly people in the United States, or 60.4% of people under age 65, used employer-sponsored health insurance in 2023.^1^ In 2024, health insurance premiums for families averaged nearly $27,000, a 7% increase from the preceding year.^2^ Continually increasing healthcare costs can limit wage growth,^3^ harming employees and exacerbating economic inequality.^4^ More than half (54%) of employee wage gains between 2000 and 2020 were estimated to be absorbed by the rising cost of benefits, primarily for health insurance.^5^

High medical costs are driven by multiple factors, including ones attributable to provider decisions such as encounter volume and service intensity.^6^ Including high-cost providers in health plans raises costs for everyone. As Rosen Hotels has demonstrated for decades, it is possible to reduce costs by providing on-site primary care and eliminating access to higher cost providers that do not offer quality advantages.^7^ In part because of the blowback from the implementation of managed care, employers have been reluctant to limit employee choice of provider.

There have been surprisingly few investigations of the trade-offs in wages and working conditions employees are willing to make in exchange for richer health benefits.^8-10^ In this paper, we report results from a national survey that examines these choices among Americans who receive health insurance through their employment. The study used volunteers who self-selected into the sample frame rather than a true random sample. The sample, however, is demographically well-matched to the employed population of the United States, and the survey methodology has been shown to predict outcomes for measurable events.^11^ For further discussion of limitations, see Supplemental Material.

## Methods

### Main survey

Data from 1340 employed respondents were collected through YouGov. The frame was constructed by stratified sampling from the full 2019 American Community Survey (ACS) one-year sample with selection within strata by weighted sampling with replacements (using the person weights from the public use file) and is representative of employed U.S. adults. Estimated propensity scores in the frame were grouped into deciles. The weights were then post-stratified on the 2016 and 2020 Presidential vote choice, and a four-way stratification of gender, age (4 categories), race (4 categories), and education (4 categories), to produce a final weighted sample of 1200 participants. A partial replication that was completed 2 months after the original survey is reported in the Supplemental Material.

### Statistical significance testing

Standard methods for sample proportions were employed to compute estimates and corresponding 95% confidence intervals for responses to survey questions. Factors associated with respondent sentiment were assessed using logistic regression. The analysis was completed using Crunch IO Software and R version 4.2.1.

### Employer survey

To offer a comparison between employee and employer perspectives, we report selected results from an employer survey conducted by Singer, Pfeffer, and colleagues, methods and results from which have been previously reported.^12,13^ Their survey included 221 nationally representative U.S. employers with at least 50 employees. Two questions allow comparison: (1) Regarding strategies for ensuring value of health benefits, does your company offer a health plan option that eliminates doctors and/or hospitals with higher-than-average charges unless there is proof that they provide higher than average quality of care; and (2) Does your company give 100% of savings from health plan options that cost less to employees that choose this option?”

## Results

### Representativeness of sample

All participants (100%) were employed by companies that offered health insurance. Among these, 46.3% reported that their employers offered insurance to employees through a private (or corporate) exchange. The mean monthly family payroll deduction for health insurance was $366 (median $200) for the 77.4% of employees who provided this information. Demographic characteristics of the 1200 participants closely matched those of the employed nonagricultural working population of the United States, using data available from the U.S. Bureau of Labor Statistics and the U.S. Census Bureau (Table 1). Gender was matched within less than 1%. One-third (33%) of the respondents had dependents under the age of 18 in the household. Self-reported income closely matches that of the employed population in 2022. (See Supplemental Material).^14^

**Table 1:** Comparison of the study sample to the US employed workforce.

	Employed workforce	YouGov sample
**Gender**		
Male	50.1%	50.8%
Female	49.9%	49.2%
**Race/Ethnicity**		
White	67.0%	64.2%
Black or African-American	12.1%	11.3%
Hispanic or Latino	17.6%	15.1%
Asian or Asian-American	5.9%	3.4%
Multiracial/Other		6.0%

Key findings are summarized in Table 2. A summary of logistic regression results that probes demographic and attitudinal correlates of item responses is shown in the Supplemental Material.

**Table 2:** Summary of responses to individual items on employee survey.

Item	Consumer survey question	Response option	Sample %	95% CI	
				Lower	upper
Satisfaction with Benefits					
Q14	Your compensation package, given in exchange for your work, is typically a combination of salary and benefits. For example, you are paid wages, and you might receive benefits like health insurance. Although you pay income tax on wages, benefits are usually not taxed. If you had a choice, would you prefer to:	Take more money in salary and reduce benefits, even though it would result in higher taxes	16.5%	14.5%	18.8%
		Keep the current balance of salary and benefits the same	49.9%	47.1%	52.8%
		Take less money in salary in exchange for more tax-free benefits	19.3%	17.1%	21.6%
		Do not know	14.3%	12.4%	16.5%
Acceptance of Narrow Networks					
Q15	Some health insurance plans restrict the doctors and hospitals you can use. This is often called a “narrow network.” An employer who selects a narrow network plan may save as much as 15% in what they spend on health insurance. Under what circumstances would you be willing to accept a health insurance plan that restricted you to certain doctors or health care facilities? (check all that apply)	If I received higher pay in exchange for using a less expensive health insurance plan	45.9%	43.1%	48.8%
		If the savings were used to support another employee benefit (like child care, vision, or dental care)	30.9%	28.3%	33.6%
		If reduced health care spending made the company more viable and less likely to lay off workers	14.6%	12.7%	16.7%
		I would not accept a narrow network plan under any circumstances	31.7%	29.1%	34.4%
Choice of Lower Cost Providers					
Q10.1	Managers of employee health benefits plans should: Offer a health insurance plan option that eliminates doctors and/or hospitals with above-average cost to patients unless there is proof that they provide above-average quality of care	Strongly agree	44.0%	41.2%	46.9%
		Agree			
		Neutral	35.2%		
		Disagree	11.7%		
		Strongly disagree	9.2%		
Disbursement of Savings					
Q10.4	Managers of employee health benefits plans should: Give 100% of any cost reductions to employees	Strongly agree	72.0%	69.3%	74.5%
		Agree			
		Neutral	20.4%		
		Disagree	3.8%		
		Strongly disagree	3.8%		
		skipped	0.1%		
Employer Trust					
Q13	To what extent do you trust your employer to negotiate for a health insurance plan that is in your best interest?	I have a *high level of trust* that my employer will negotiate on my behalf	74.8%	72.2%	77.2%
		I have a *moderate level of trust* that my employer will negotiate on my behalf.			
		I have a *moderate level of distrust* that my employer will negotiate on my behalf	16.9%		
		I have a *high level of distrust* that my employer will negotiate on my behalf	8.3%		
Employer Responsibility					
Q17	Should your employer be held accountable for any problems that arise as a consequence of the health insurance and healthcare you receive?	Yes	30.6%	28.0%	33.3%
		No	44.3%		
		Not sure	25.2%		

Authors’ analysis of a June 2022 YouGov survey of 1200 US adults using employer-sponsored insurance (ESI).

About half (49.9%, 95% CI: 47.1%, 52.8%) of our respondents with employer-sponsored health benefits reported a preference for keeping their current balance of benefits and salary (Q14). Because of the well-known status quo bias, a preference for maintaining present arrangements does *not* necessarily reflect satisfaction with those benefits.^15^ Only 16.5% (95% CI: 14.5%, 18.8%) of respondents reported they would “take more money in salary and reduce benefits, even though it would result in higher taxes.” These respondents tended to be younger (OR 1.05, *P* < 0.001) and male (OR 1.78, *P* = 0.001) (Supplemental Material).

Employees varied in their consideration of plans with narrow provider networks (Q15). Among respondents, 31.7% (95% CI: 29.1%, 34.4%) “would not accept a narrow network plan under any circumstances.” These respondents tended to be older (OR 0.96, *P* < 0.001) and less likely to have children under 18 (OR 0.63, *P* = 0.002) (they were also less trusting of employers to negotiate on behalf of employees, OR 0.69, *P* = 0.013). Conversely, 68.3% of the respondents would be willing to accept a health insurance plan that restricted access to certain doctors or health care facilities under some circumstances. For instance, 45.9% (95% CI: 43.1%, 48.8%) would accept a narrow network if they were to receive “higher pay in exchange for using a less expensive health insurance plan.” A narrower network would be accepted by 30.9% of the respondents (95% CI: 28.3%, 33.6%), “if the savings were used to support another employee benefit (like childcare, vision, or dental care).” Those who might accept a narrow network for additional benefits tended to be younger (OR 1.02, *P* < 0.001), liberal (OR 1.72, *P* = 0.001), and more trusting of their employer to negotiate on their behalf (OR 1.58, *P* = 0.003).

44.0% of respondents (95% CI: 41.2%, 46.9%) agreed or strongly agreed that managers of employee health benefits plans should “offer a health insurance plan option that eliminates doctors and/or hospitals with above-average cost to patients unless there is proof that they provide above-average quality of care” (Q10.1). Importantly, employee perspectives on whether higher cost providers should be excluded (without evidence of higher quality of care) did not differ based on respondent age, gender, and responsibility for children under the age of 18. Black or African-American individuals, more than White individuals, and those self-identifying as liberals expressed greater agreement that employers should offer a plan option that eliminates higher cost providers absent evidence of higher quality care (OR 2.03, *P* < 0.001 and OR 2.05, *P* < 0.001, respectively).

Meanwhile, Singer et al.^13^ and Pfeffer et al.^12^ found that only 10% of employers with at least 50 employees indicated that they “offer a health plan option that eliminates doctors and/or hospitals with higher-than-average charges unless there is proof that they provide higher than average quality of care.”

Employees overwhelmingly expressed a preference for being the beneficiaries of the health plan savings. 72.0% (95% CI: 69.3%, 74.5%) said that managers of employee health plans should give 100% of any cost reductions to employees (Q10.4). These respondents are more likely to identify as liberal (OR 1.99, *P* < 0.001). Our follow-up replication (see Supplemental Material) indicated that respondents did not differentiate between giving 100% of savings to employees who choose a lower-cost plan that eliminates high-cost providers vs giving the savings to employees regardless of plan choice (X^2^ = 5.7, *P* = 0.22).

Three-quarters of respondents (74.8%, 95% CI: 72.2%, 77.2%) trusted their employer to negotiate for a health insurance plan that is in the best interest of employees (Q13). While results indicated elevated trust among those born prior to 1960, we did not find significant associations with most other respondent characteristics.

Almost a third, some 30.6% of respondents (95% CI: 28.0%, 33.3%), reported employers should be held accountable for any problems that arise as a consequence of the health insurance and healthcare their employees receive (Q17). Respondents who endorsed this statement tended to be significantly younger (OR 1.03, *P* < 0.001), male (OR 1.64, *P* < 0.001), Black or African-American (OR 1.81, *P* = 0.004), have children under 18 (OR 1.61, *P* = 0.001), and liberal (OR 1.84, *P* = 0.001).

## Discussion

Health insurance benefits are often seen as a benevolent contribution by employers, and they are rarely framed as earned compensation. Yet, in exchange for higher benefits, workers typically receive lower wages and sometimes poorer working conditions.^16^ Data from the KFF 2025 Employer Benefits Survey suggest that approximately $27 000 of the compensation package of American workers is devoted to health insurance premiums.^2^

Employers make health benefits decisions on behalf of their employees, which, under the Employee Retirement Income Security Act (ERISA), creates a fiduciary responsibility to act in the employees’ interests. ^17^ Using survey data from a representative sample of U.S. employers, Singer et al.^13^ recently demonstrated that the majority of companies neither measured key indicators of health benefits performance nor assigned responsibility for managing them.

Our study reveals that consumers of employer-sponsored health benefits want plan options that allow trade-offs between provider breadth and cost. Employees also expect to retain savings associated with choosing narrow network plans through higher pay or benefits. Yet few large employers offer lower-cost networks that return the full savings to employees. The survey of employers revealed that only 16% return 100% of savings to employees.^12^ Future research might ask employees how much they value specific plan attributes and then test alternative configurations.

Not surprisingly, employee preferences varied by age, sex, race, parental status, and ideology. This variation underscores the importance of offering at least 1 option that allows employees to save money by choosing a narrower network and to see those savings reflected in their paychecks. Higher salaries could result from passing along premium savings and cost reductions driven by plan design. We recognize that how savings from narrower networks are passed on is likely to be more complex than the survey framing suggests.

Our results are consistent with several other investigations into employee preferences. In the late 1990s, Enthoven and associates documented consumer backlash against managed care and called for greater consumer choice.^18^ Ullman and colleagues, using data from New York City, Dallas, Houston, and Washington, D.C., demonstrated that consumer satisfaction was more strongly associated with the ability to select a plan than with having a choice at the point of service.^19^ A KFF/Los Angeles Times poll of adults with employer-sponsored insurance found 4 in 10 families had problems with affordability, 6 in 10 chose cost-related factors (low premiums, deductibles, or co-pays) as the most important feature in a health plan, while about a quarter chose coverage-related factors (choice of providers or range of covered benefits).^20^

Other evidence suggests that narrow networks may not always be the most effective mechanism for lowering costs because of deficiencies in price negotiation. Wing and colleagues found that cash prices for shoppable services often reflected larger discounts than negotiated prices.^21^ Similarly, Jiang, Makary, and Bai showed that cash prices were frequently lower than payer-negotiated prices. We recognize that employers are sometimes limited in their ability to negotiate prices, particularly given consolidation among third-party administrators.^22^ But before asking employees to forgo provider choice, purchasers should demonstrate greater negotiating effectiveness.

## Conclusions

Employers design compensation packages to attract and retain a skilled workforce. After World War II, wage and price controls limited salary increases, leading employers to introduce health insurance as a pre-tax benefit. This shift moved compensation from wages to benefits, with employees indirectly funding coverage through lower salaries.

Despite the central role of employer-sponsored insurance, little attention has been paid to employee preferences in health plan design. Our study suggests that employees want to share in the financial benefits of lower-cost plans and are willing to limit provider choice to reduce costs. Greater attention to employee preferences could improve benefit design, enhance employee satisfaction, and better align compensation with workforce priorities.

## Supplementary Material

qxag120_Supplementary_Data
