# Supplementary material for: Employee preferences in health plan design: results from a national survey
Source: Health Aff Sch. 2026 Jun 19;4(6):qxag120. doi: 10.1093/haschl/qxag120 (PMC13282439; doi:10.1093/haschl/qxag120)
Supplement: qxag120_Supplementary_Data [file qxag120_supplementary_data.zip › Employee Survey Suplemental Materials 6-3-2026_mcn-bk.docx]

Supplemental Materials for Health Affairs Scholar: Employee Preferences in Health Plan Design: Results from a National Survey

Appendix 1

Table of Contents.

Item Page

Screening Questions 2

Follow-Up Replication Study 3

Limitations 5

Appendix Table A1: Characteristics of Survey 7

Respondents (Main Survey)

Appendix Table A2: Summary of Responses to 8

Individual Items on Follow-Up Employee Survey

Appendix Table A3: Results of Logistic 9

Regression Analysis (Main Survey)

**Screening Questions**

Do you have health insurance through a current or former employer or union (yours or another family member's)?

Do you have health insurance purchased directly from an insurance company (yours or another family member's)?

Do you have health insurance from Medicare for people 65 and older or with certain disabilities?

Do you have health insurance from Medicaid, Medical Assistance, or any government assistance plan for those with low incomes or a disability?

Do you have TRICARE or other military health care?

Do you have health insurance through the VA (enrolled for VA health care)?

Do you have health insurance through the Indian Health Service?

Do you have any other type of health insurance or health coverage plan?

Does your organization offer health insurance to its active employees?

How many different health insurance plans are offered by your employer?

Does your employer offer health insurance through a private exchange?

Does your employer offer other health benefits?

Dental

Vision

Mental Health

**Follow-Up Replication Study**

Two months after the original survey, three related questions were included in a nationally representative YouGov omnibus survey. Using the same methods as in the main survey, propensity scores were used to select 1,200 individuals that maximized the match between the study sample and the US Employed population on age, sex, race, and education. The selection criteria for participants were identical to those used in the original study. We refer to this exercise and the “Follow-Up Survey”.

One question from the initial survey was repeated: whether employers should offer a health insurance plan option that eliminates doctors and/or hospitals with higher than average charges unless there is clear evidence that they provide higher than average quality of care. Two new items were added. Both asked respondents to indicate their level of agreement with the idea of giving 100% of health insurance savings back to employees. One item specified that the savings should go to employees who select lower-cost plan options, while the other proposed sharing savings from any employer efforts to reduce overall health insurance spending. Unlike the original survey, the follow-up reversed the polarity of the Likert scale. In the original survey, response options ranged from Strongly disagree to Strongly agree. In the replication, Strongly agree appeared first, and Strongly disagree was listed last. Data from the main survey and data from the follow-up survey were analyzed separately; results from the follow-up employee survey are shown in Appendix Table A2.

The follow-up survey, which included just three questions that probed consumer preferences regarding employer-sponsored health benefits in the national YouGov omnibus survey, revealed that 52.8% of respondents (95% CI: 49.9%, 55.6%) agreed that managers of employee health benefits plans should “offer a health insurance plan option that eliminates doctors and/or hospitals with higher than average charges unless there is proof that they provide higher than average quality of care” (S2_Q10.1). This was significantly greater than the 44.0% agree rate in the original survey (Chi^2^ test statistic = 18.0, *p* < 0.001).

The follow-up survey found that 59.5% of respondents (95% CI: 56.7%, 62.3%) agreed that managers of employee health benefits plans should “give 100% of savings from [a less expensive] health insurance plan option to employees that choose this option” (S2_Q10.2). Additionally, the follow-up survey showed that 55.6% of respondents (95% CI: 52.7%, 58.4%) agree that managers should “give 100% of savings to employees from all other employer efforts to lower health insurance plan spending” (S2_Q10.3). The percentage of respondents who disagreed or strongly disagreed with these statements was 8.4% in both cases.

Limitations

Our results should be interpreted in light of several limitations. Importantly, the methodology is not a true random sample of employees. The YouGov methodology uses volunteers who self-select into the sample frame. Then, the sample is crafted to match the general population based on a weighting methodology. Although the method is not a true random sample, it is demographically well-matched to the target population -- in this case, the employed population of the United States. Further, the methodology has been shown to predict outcomes for measurable events.^14^ For example, using this method, YouGov has been very accurate in predicting the popular vote in U.S. elections.

The fact that our entire sample had employer-sponsored health insurance is both a strength and a limitation. Research indicates that individuals without health insurance are more likely to have lower incomes, belong to minority racial or ethnic groups, and experience poorer health outcomes.^15^ Additionally, focusing solely on employees with private insurance limits the generalizability of our findings to the significant portion of the U.S. population that receives care through public programs such as Medicare, Medicaid, and the Veterans Administration. One study analyzing data from nearly 150,000 respondents to the Behavioral Risk Factor Surveillance System found that individuals with private insurance had less access to healthcare, faced higher costs, and reported lower satisfaction with services compared to those covered by public insurance options.^16^

We recognize that employers use other mechanisms to get feedback from employees. Unfortunately, we do not have access to this information which is often proprietary and confidential. Another limitation is that we do not know what data o employers have in mind when making benefit decisions, how employers benchmark plan offerings within the labor market, and what feedback mechanisms employers do use. Future research should consider what groups of employees and employers have in mind when making benefit decisions, and how employers benchmark plan offerings within the labor market. We recognize that an employer’s ability to craft targeted networks may be limited.

Another important limitation relates to asking employees to evaluate employer decisions about provider networks. Many workers do not know whether their plan is narrow-network, which hospitals or physician groups are included, or the rationale for inclusion. For example, an employee would have no way to know whether Stanford is in-network because of price, quality, contracting leverage, or other factors. Moreover, stated preferences may not match real-world choices: people may endorse “high-quality networks” in principle, but react differently when a preferred referral site is out-of-network. These tensions suggest the survey does not fully capture the complexity of actual trade-offs. Our study assessed one of many possible tradeoffs, network design; further research should assess multiple plan attributes and alternative plan configurations in order to better evaluate employee perspectives.

It is important to use caution in comparing results from this YouGov survey of employees with the Singer/Pfeffer et al. survey of employers. Although the two studies used some of the same questions, the sampling strategies for the surveys differed, and the employees we studied did not work for the firms studied in the employer survey.

Appendix Table A1: Characteristics of Survey Respondents (Main Survey)

Appendix Table A2: Summary of Responses to Individual Items on Follow-Up Employee Survey

Appendix Table A3: Results of Logistic Regression Analysis (Main Survey)
